# Antireflective SiC Surface Fabricated by Scalable Self-Assembled Nanopatterning

**DOI:** 10.3390/mi7090152

**Published:** 2016-09-01

**Authors:** Yiyu Ou, Ahmed Fadil, Haiyan Ou

**Affiliations:** Department of Photonics Engineering, Technical University of Denmark, Ørsteds plads 343, Kongens Lyngby DK-2800, Denmark; yiyo@fotonik.dtu.dk (Y.O.); afad@fotonik.dtu.dk (A.F.)

**Keywords:** rapid thermal process, self-assembled nanopattern, antireflective surface, sub-wavelength structure, silicon carbide

## Abstract

An approach for fabricating sub-wavelength antireflective structures on SiC material is demonstrated. A time-efficient scalable nanopatterning method by rapid thermal annealing of thin metal film is applied followed by a dry etching process. Size-dependent optical properties of the antireflective SiC structures have been investigated. It is found that the surface reflection of SiC in the visible spectral range is significantly suppressed by applying the antireflective structures. Meanwhile, optical transmission and absorption could be tuned by modifying the feature size of the structure. It is believed that this effective fabrication method of antireflective structures could also be realized on other semiconductor materials or devices.

## 1. Introduction

Over the past decades, technology for energy-efficient devices such as solar cells and light-emitting diodes (LEDs) has developed rapidly due to the upcoming energy shortage [1,2]. It is of crucial importance to enhance device efficiency in order to expand the application range and improve the cost effectiveness. Due to the large refractive index discontinuity at the air–semiconductor material interface, solar cell devices have a large surface reflection [1,3] and LEDs usually suffer from low light extraction efficiency because of the total internal reflection [2,4]. A broadband antireflection or light extraction improvement can be achieved by applying a stack of antireflection coatings with an appropriate design [5,6]. However, this material system is limited by the availability of materials with suitable refractive indices and thermal expansion coefficients. Using sub-wavelength nanostructures has been extensively reported as an effective way to reduce the surface reflection on solar cells [1,3,7] or to enhance the light extraction on LEDs [2,4,8]. In order to fabricate the nanostructures, a nanopatterning process such as e-beam lithography [9,10], nanoimprint lithography [3,11], or nanospheres lithography [12,13] is usually indispensable to create a mask layer for the subsequent etching process. In addition, applying a rapid thermal process (RTP) to a thin metal film (such as Au, Ag or Ni) is proven as a time-saving and scalable method to create a nanopattern with a controllable feature size. Self-assembled nanoparticles formed this way can be applied on samples from chip size to wafer size and it has been widely used on GaN [14], Si [15], and other semiconductor materials [16,17,18].

In this work, we demonstrated nanopattern formation by conducting RTP to thin Au films on a SiC substrate which is a promising material for both solar cell and LED applications [19,20]. The self-assembled metal nanoparticles with a controlled feature size and structure density were investigated. Followed by a dry etching process, nanostructures were formed on the SiC surface. In addition, size-dependent optical properties of nanostructures were also studied.

## 2. Fabrication Process Flow

**Figure 1:**
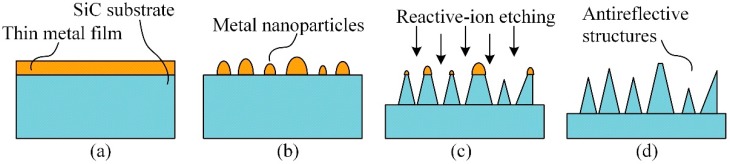
Schematic illustrations of the sub-wavelength antireflective structure fabrication steps: (**a**) Thin metal film deposition; (**b**) Rapid thermal process to form the nanopatterns; (**c**) Dry etching process; (**d**) Removal of residual mask.

The fabrication process of sub-wavelength antireflective structures on a SiC substrate is schematically illustrated in Figure 1. The process consists of two main parts: nanopatterning and dry etching. Firstly, a thin metal film (Au) was deposited on the SiC surface by using the e-beam evaporation process (Figure 1a). To form the nanopattern, the sample was treated by RTP at 650 °C for 3 min in N_2_ ambient and the thin Au film on the SiC surface was agglomerated into nanoparticles which minimized the surface energy (Figure 1b). Thereafter, a reactive-ion etching (RIE) process with SF_6_ and O_2_ plasmas was applied and the sub-wavelength antireflective structures were formed on the SiC surface by using the Au nanoparticles as a mask layer (Figure 1c). Finally, the residual Au nanoparticles were removed by using an iodine-based solution (Figure 1d).

## 3. Nanopatterning

The nanoparticles formed in the RTP step function as the mask pattern during the dry etching process and therefore the feature size and structure density of the formed antireflective structures were mainly determined by the corresponding values of the nanoparticles. Nanoparticles with different density and structure size were achieved by varying the thickness of the deposited thin metal film. Six samples named from a to f were prepared with the deposited Au film thickness ranging from 3 to 13 nm in a step of 2 nm, respectively. The thickness of the Au film was well controlled during the e-beam evaporation process with a low deposition rate of 1 Å/s for all the samples.

**Figure 2:**
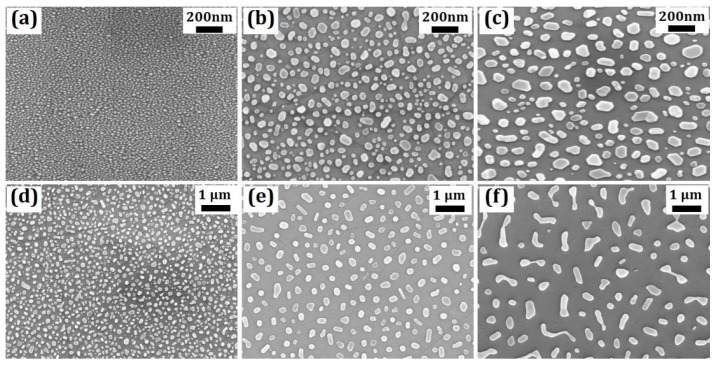
Top-view SEM image of Au nanoparticles formed by rapid thermal process (RTP) of samples with varied deposited Au film thicknesses: (**a**) 3 nm; (**b**) 5 nm; (**c**) 7 nm; (**d**) 9 nm; (**e**) 11 nm and (**f**) 13 nm, respectively.

Although the self-assembled nanoparticles formed by the RTP method do not have a uniform size distribution, the average size of the structure can be controlled by tuning the thickness of the deposited metal film in a certain range. Figure 2 shows the top-view scanning electron microscopy (SEM) images of Au nanoparticles formed from samples with different Au film thicknesses. It is shown that the average size of the nanoparticles increases with the thickness of the Au film. Meanwhile, the structure density in a unit area decreases. However, for sample f with the largest Au film thickness of 13 nm, large connections start to appear between the nanoparticles and so the structure uniformity shows worse performance compared to samples made from relatively thin Au film.

**Figure 3:**
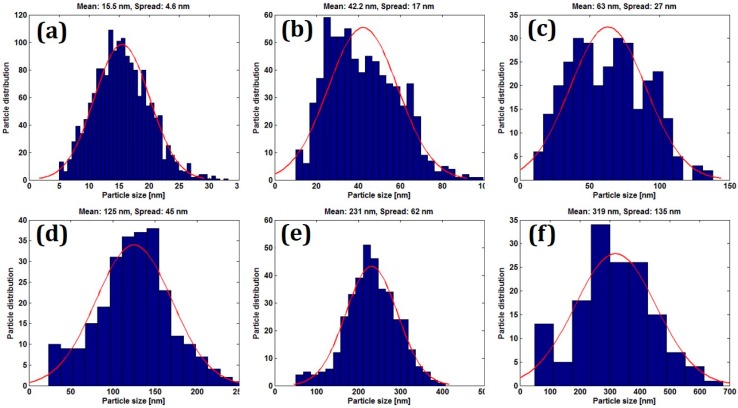
(**a**–**f**) Size distribution of nanoparticles formed by RTP of samples with deposited Au film thicknesses of 3 nm, 5 nm, 7 nm, 9 nm, 11 nm and 13 nm, respectively.

A detailed study of nanoparticle structure dependence on Au film thickness was conducted. From the top-view SEM images, the information of the nanoparticle size distribution, nanoparticle density and nanoparticle coverage can be obtained by using normal graphical software. The size distributions of the nanoparticles were presented in Figure 3. Here we define the size of each nanoparticle as the diameter of its bottom area, and the size distribution in different samples was analyzed based on this assumption. The size of the Au nanoparticle roughly follows a Gaussian distribution for most of the samples and the average nanoparticle size increases generally with the Au film thickness as we can see from the top-view SEM images shown in Figure 2. Meanwhile, the size standard deviation (spread/mean) also increases from 29.7% for sample a (3 nm Au) to 42.3% for sample f (13 nm Au) which indicates a better size uniformity for nanoparticles made from a thinner Au film.

Table 1 gives the statistics of Au nanoparticle structures in terms of the Au film thickness, structure density, coverage, height, and aspect ratio, where “nanoparticle density” is the number of nanoparticle structures per unit area, “nanoparticle coverage” is the ratio of the area covered by nanoparticles to the total area of the sample, “nanoparticle height” is the calculated average height of the nanoparticle (defined as the ratio of the Au film thickness to the nanoparticle coverage), and “nanoparticle aspect ratio” is the ratio of the structure height to the bottom size. One can see that the nanoparticle density decreases with the increased Au film thickness and this is in accordance with the observed increase in the structure size. A similar trend is also noted in the variation of the nanoparticle coverage. By knowing the Au film thickness before RTP and the nanoparticle structure coverage after RTP, the average nanoparticle height was deduced accordingly. Hence, the average aspect ratio of the nanoparticle structure is also calculated.

From the above discussion, it is seen that nanoparticles formed by RTP of thin Au film is an effective way to create a nanopattern with a controllable average feature size from 15 to 319 nm, simply by tuning the thickness of the deposited thin film. It is also found that, with the increased Au film thickness, the nanoparticle has a larger structure size as well as an increased structure height. Meanwhile, the area coverage by the nanoparticle structure decreases and the structure tends to get a lower aspect ratio. 

## 4. Dry Etching and Characterization

To achieve an antireflective surface, sub-wavelength structures were fabricated on a 6H-SiC substrate by applying the RIE process with Au nanoparticles as the etching mask. During the dry etch process, a mixture of SF_6_ and O_2_ plasmas was used as a reactive gas under the optimized chamber conditions. Since the patterned nanoparticles have different sizes, a longer etching time was applied on samples made from thicker Au film in order to achieve a cone-shape structure with better antireflective performance. Other conditions were maintained the same during the dry etch process.

**Figure 4:**
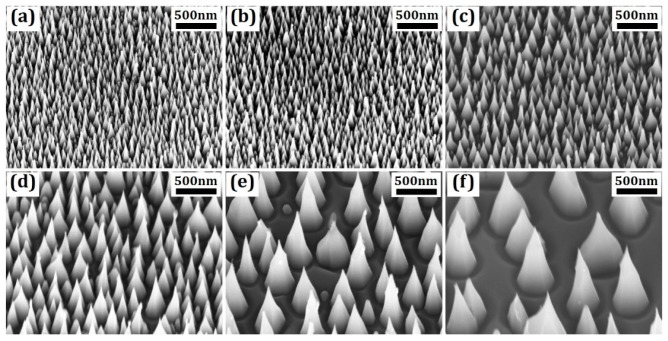
(**a**–**f**) SEM image of antireflective structures on SiC substrate fabricated from samples with deposited Au film thicknesses of 3 nm, 5 nm, 7 nm, 9 nm, 11 nm, and 13 nm, respectively.

Fabricated antireflective structures are shown in Figure 4. It can be seen that cone-shape nanostructures were achieved on all the samples while the bottom sizes of the structures are mainly determined by the size of the nanoparticles as a mask layer. The measured structure height is in the range of 83–245 nm for sample a (3 nm Au), and the height increases with the Au film thickness due to a longer etching time. Sample f (13 nm Au) has a cone structure height of 494–1040 nm. It is also seen that the larger nanostructure has a more irregular shape which can be attributed to less uniformity of the nanoparticles formed from thick Au films.

**Figure 5:**
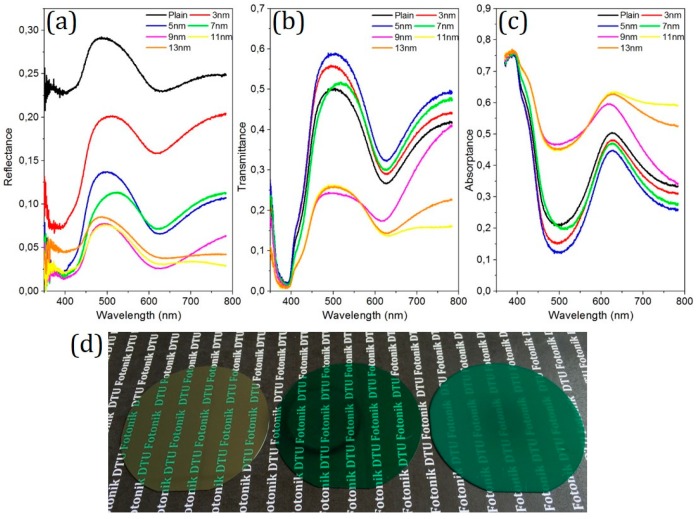
(**a**) Reflectance; (**b**) Transmittance; (**c**) Absorptance of different antireflective structures over the visible spectral range; (**d**) Photo images of 2 inch SiC substrates (from left to right: with plain surface (reference sample), largest transmittance (sample b: 5 nm Au) and lowest reflectance (sample e: 11 nm Au)).

Optical properties were studied on the nanostructures in terms of surface reflectance and transmittance over the visible spectral range. The measurements were conducted by using a calibrated integrating sphere system (OL 700-71, Gooch & Housego, Ilminster, UK) together with an optical spectrometer (CAS 140B, Instrument Systems Inc., München, Germany) to obtain the total surface reflectance and transmittance. The results are demonstrated in Figure 5a,b. The average surface reflectance of the SiC substrate in the measured range was decreased dramatically from 25.2% of the plain surface to 4.2% of sample e (11 nm Au) by applying nanostructures on the surface. It is noted that larger nanostructures generally have a lower surface reflectance. On the other hand, the optical transmittance initially increases with the nanostructure size, and then drops and becomes lower than that of the plain sample. This phenomenon indicates that reduced surface reflectance does not necessarily lead to an increased transmittance. Thereafter, optical absorptance was achieved and the results are shown in Figure 5c. Nanostructures with a relatively small size (samples a–c) have a lower absorptance compared to the plain sample which is ascribed to the increased transmittance/light extraction and reduced internal reflection. In contrast, larger nanostructures (samples d–f) have a larger surface area and possibly more surface damage introduced during the dry etching process. It is believed that such surface states function as trapping centers and therefore increase the optical absorptance. Figure 5d shows the photo images of 2 inch SiC substrates with a plain surface (reference sample), the largest transmittance (sample b) and the lowest reflectance (sample e), respectively. It is clearly seen that, with different nanostructures on the SiC substrate, a significant color change of the SiC wafer surface can be observed by the naked eye.

## 5. Conclusions

In summary, a time-efficient nanopatterning method by RTP applied to a thin metal film has been demonstrated on 2 inch SiC wafer substrates. Nanoparticle structures formed in this way have a tunable average size ranging from 15 to 319 nm. Not only the structure size but also the structure density and area coverage can be controlled within a certain range. Followed by a dry etching process, nanostructures can be achieved on the SiC substrate surface with strong antireflection performance. For nanostructures with relatively small size, the surface reflectance decreases; meanwhile, transmittance increases due to the reduced internal reflection. Larger nanostructures have a better antireflection performance which is mainly due to the enhanced optical absorption from the surface caused by the increased amount of surface states. As a result, by designing the feature size of the nanopattern well, the optical properties of nanostructures can be tuned in terms of optical reflectance, transmittance and absorptance. Such a scalable nanopatterning method is quite a promising technique which could also be applied on other optoelectronic materials or devices.

## Figures and Tables

**Table 1:** Statistics of nanoparticle structures patterned from rapid thermal process (RTP) of thin Au film in terms of Au film thickness, structure density, coverage, thickness, and aspect ratio.

Au Film Thickness (nm)	Nanoparticle Density (/µm^2^)	Nanoparticle Coverage (%)	Nanoparticle Height (nm)	Nanoparticle Aspect Ratio
3	1910	39.2	7.7	0.50
5	200	32.3	15.5	0.37
7	88.9	32.8	21.3	0.33
9	19	26.4	34.1	0.27
11	4.3	19.4	56.7	0.25
13	1.7	16.3	79.8	0,25
